# Switching on Endogenous Metal Binding Proteins in Parkinson’s Disease

**DOI:** 10.3390/cells8020179

**Published:** 2019-02-19

**Authors:** Fleur A. McLeary, Alexandre N. Rcom-H’cheo-Gauthier, Michael Goulding, Rowan A. W. Radford, Yuho Okita, Peter Faller, Roger S. Chung, Dean L. Pountney

**Affiliations:** 1 School of Medical Science, Griffith University, Southport, QLD 4222, Australia; f.mcleary@griffith.edu.au (F.A.M.); alex.rcom@hotmail.com (A.N.R.-H.-G.); michael.goulding@griffithuni.edu.au (M.G.); yuho.okita@gmail.com (Y.O.); 2 Centre for Motor Neuron Disease Research, Macquarie University, Department of Biomedical Sciences, Faculty of Medicine & Health Sciences, Sydney, NSW 2109, Australia; rowan.radford@hdr.mq.edu.au (R.A.W.R.); roger.chung@mq.edu.au (R.S.C.); 3 Institut de Chimie, UMR 7177, CNRS-Université de Strasbourg, 4 rue Blaise Pascal, 67000 Strasbourg, France; pfaller@unistra.fr

**Keywords:** copper, iron, calcium, alpha-synuclein, Parkinson’s disease, metallothionein, calbindin, ferritin

## Abstract

The formation of cytotoxic intracellular protein aggregates is a pathological signature of multiple neurodegenerative diseases. The principle aggregating protein in Parkinson’s disease (PD) and atypical Parkinson’s diseases is α-synuclein (α-syn), which occurs in neural cytoplasmic inclusions. Several factors have been found to trigger α-syn aggregation, including raised calcium, iron, and copper. Transcriptional inducers have been explored to upregulate expression of endogenous metal-binding proteins as a potential neuroprotective strategy. The vitamin-D analogue, calcipotriol, induced increased expression of the neuronal vitamin D-dependent calcium-binding protein, calbindin-D28k, and this significantly decreased the occurrence of α-syn aggregates in cells with transiently raised intracellular free Ca, thereby increasing viability. More recently, the induction of endogenous expression of the Zn and Cu binding protein, metallothionein, by the glucocorticoid analogue, dexamethasone, gave a specific reduction in Cu-dependent α-syn aggregates. Fe accumulation has long been associated with PD. Intracellularly, Fe is regulated by interactions between the Fe storage protein ferritin and Fe transporters, such as poly(C)-binding protein 1. Analysis of the transcriptional regulation of Fe binding proteins may reveal potential inducers that could modulate Fe homoeostasis in disease. The current review highlights recent studies that suggest that transcriptional inducers may have potential as novel mechanism-based drugs against metal overload in PD.

## 1. Parkinson’s Disease and Alpha-Synuclein

**Figure 1:**
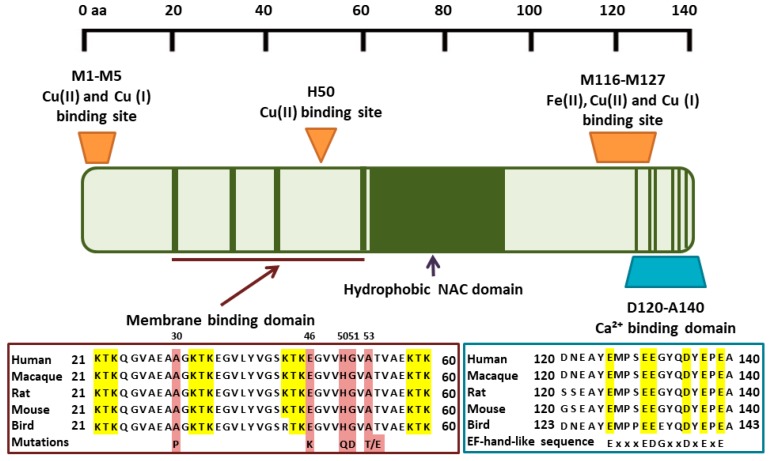
Metal binding to α-synuclein. Cu(I)/(II) binds to high-affinity N-terminal and lower affinity C-terminal sites. Ca(II) binds with low affinity to acidic residues in the C-terminus, overlapping with the low affinity Cu(I)/Cu(II) and Fe(II) binding sites. Mutated residues in familial PD are highlighted in brown. KTK repeats in the vesicle membrane binding domain are highlighted in yellow. Conserved residues in the C-terminus thought to mediate Ca binding are highlighted in yellow.

Parkinson’s disease (PD) is a complex and progressive neurodegenerative disease which affects >5 million people, with >85% of cases being sporadic with no known cause. It becomes more prevalent with age, affecting 2% of those over 60 and 3% of those over 80. Debilitating symptoms include tremors, movement and balance issues, difficulty swallowing and speaking, rigid muscles, depression, anxiety, cognitive impairment, and dementia [1,2,3]. These symptoms are largely caused by the progressive loss of dopamine-secreting neurons in the substantia nigra (Sn). Diagnosis is generally only possible in the later stages of the disease because symptoms only start to arise after up to 80% of neurons have already been lost [4]. Existing drugs provide only symptomatic relief of some of these symptoms and often have severe side effects (there are no disease-modifying therapies demonstrated to slow/stop PD progression), while the degenerative nature of the disease continues unchecked with existing symptoms worsening and new symptoms arising. The accumulation of primarily neuronal protein inclusion bodies called Lewy bodies (LB) is the main pathological hallmark of PD. The normally pre-synaptic vesicle-associated protein, α-synuclein (α-syn), is the main protein component of LB, with α-syn in Lewy bodies being largely in the form of filamentous aggregates and phosphorylated at serine 129 [5]. Some 27 different gene mutations have been linked to familial PD cases, including 6 specific gene mutations in the gene encoding for α-syn, that have amino acid substitutions, A30P, A53T, E46K, G51D, H50Q, and A53E [6], although to date no successful treatments have been developed by targeting the genes identified. Further, misfolded/aggregated α-syn has also been linked both immunohistochemically and biochemically to idiopathic PD. Neuron-to-neuron transfer of α-syn aggregates between neuroanatomically connected areas of the brain is thought to be the mechanism underlying the pathological progression of PD. Multiple in vitro and in vivo studies indicate a central role for α-syn-mediated toxicity in PD pathogenesis, where toxic forms of aggregated α-syn are released from neurons, transfer between cells, and seed/template in a “prion-like” manner the endogenous α-syn in recipient neurons into a toxic aggregating form [7]. α-Syn misfolding is believed to be the most important factor driving LB formation in both familial and idiopathic cases, with many agents promoting α-syn to misfold and aggregate, including reactive oxygen species (ROS) and elevated concentrations of metals, such as Ca, Cu, or Fe [8]. Indeed, Ca, Fe, and Cu all bind α-syn at distinct metal-binding sites (Figure 1). This review will focus on the roles of Ca, Cu, and Fe in PD and related diseases, such as dementia with Lewy bodies and multiple system atrophy, and the potential to tackle dyshomeostasis of these metal ions by the induction of endogenous metal chelating proteins. Although metal dysregulation is expected to be detrimental to both neuronal and glial cell types, the discussion will deal primarily with intraneuronal and extracellular interactions involving α-syn.

## 2. Cu Dyshomeostasis in Parkinson’s Disease

There is a substantial body of evidence that Cu is dysregulated in PD [9]. Cu is needed for a multitude of processes in the healthy body and must be acquired as an essential trace element. Processes highly sensitive to adequate Cu levels include synthesis of neurotransmitters, transformation of energy within the mitochondria, antioxidant defenses, and cell signaling. There is evidence of Cu transporter-inefficacy that occurs over time that could lead to the accumulation of Cu in aged individuals [10,11,12]. This provides a central theory about Cu dyshomeostasis in PD. Cu levels are increased in PD blood serum, with high serum Cu correlated positively with disease severity [13], although a more recent study found a reduced Cu status index in PD serum [14]. Cu is also increased in cerebrospinal fluid of PD cases [15]. Total tissue Cu and neuromelanin-bound Cu, however, is decreased in the Sn of PD patients, as is expression of the Cu transporter, CTR1 [16,17,18]. Cu is regulated by transmembrane transporters, including Cu transporter 1 (CTR1) and ATP7A/B, so it is likely that decreased expression of CTR1, the main plasma membrane transporter for Cu in the brain, could be indicative of a faulty Cu transportation system in PD disease cases. Cu in the soluble fraction of brain tissue homogenate is significantly reduced in PD cases, being almost half the normal level, but Cu is increased in the insoluble fraction [19]. The varied levels of Cu detected across different tissues and fluids may be due to the improper functioning or expression of Cu transporting ATPases, which could result in the metal being unable to leave a particular region and becoming deficient in another. The Cu ATPase, ATP7A, is a cellular transmembrane Cu pump, which when genetically mutated directly causes a disease of copper deficiency in the brain (Menkes’ disease). Likewise, genetic mutation to the ATP7B transporter, which removes the metal from cells and from the body via the bile, causes a disease of Cu accumulation (Wilson’s disease) [18,20,21]. Indeed, a recent proteomics study found increased expression of ubiquitin C-terminal hydrolase L1 (UCHL-1) in human ATP7A^-/y^ fibroblasts providing a potential link between Cu dyshomeostasis and the familial PD PARK5 mutation [22]). 

**Figure 2:**
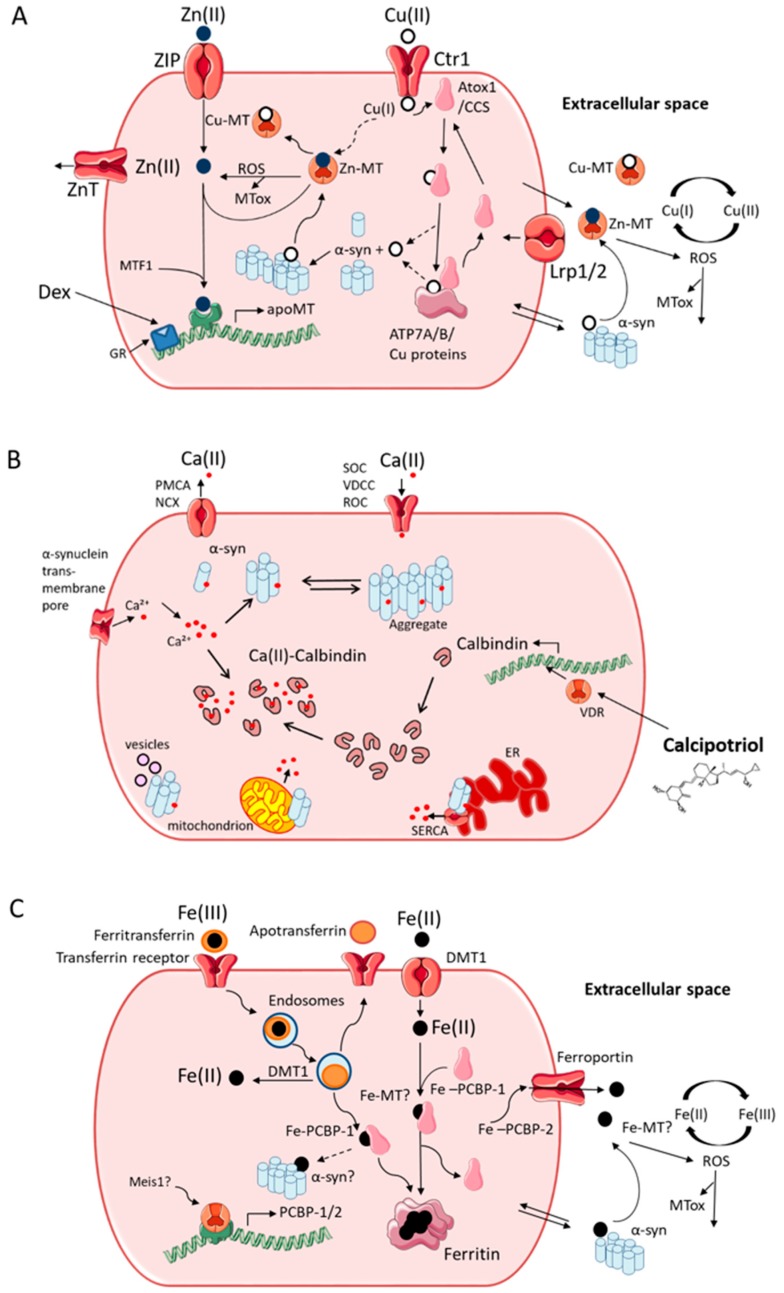
Interactions between metal ion homeostatic mechanisms and α-syn aggregation. (**A**) Cu homeostasis, α-syn and metallothionein (MT) induction. Cu enters via CTR1, then transfers to either Atox1 or CCS transporters that shuttle Cu to either ATP7A/B pumps or SOD1, respectively. MT may buffer labile Cu that spills over from these pathways. Cu binding to α-syn can induce aggregation. Zn-MT can remove Cu from α-syn. MT is also a potent ROS scavenger. Both MT and α-syn can be secreted by and taken up by cells. MT transcription is induced by MTF1 in response to Zn and by glucocorticoids via the glucocorticoid receptor. The glucocorticoid analogue, dexamethasone, induces MT and can block Cu-dependent α-syn aggregation. MTF1, metal-responsive transcription factor-1; MTox, oxidized MT; Dex, dexamethasone; GR, glucocorticoid receptor; ZnT, zinc transporter; CTR-1, copper transporter 1; CuMT, copper MT; apoMT, metal-free MT; Lrp1/2, Low density lipoprotein receptor-related protein 1/2; ZIP, zinc importer protein. (**B**) Ca homeostasis, α-syn and calbindin-D28k induction. Ca enters via ligand and voltage-gate channels and is rectified by plasma membrane pumps. Ca binding to α-syn leads to aggregation and enhances vesicle interactions. Aggregated α-syn can also allow Ca entry and stimulate Ca release from the ER and mitochondria. CB buffers intraneuronal Ca and is upregulated by the vitamin D receptor. The vitamin D analogue, calcipotriol, can induce CB and block Ca-dependent α-syn aggregation. PMCA, plasma membrane calcium channel; NCX, sodium/calcium exchanger; SOC, store-operated channel; VDCC, voltage-dependent calcium channel; ROC, receptor-operated channel; VDR, vitamin-D receptor; SERCA, sarco/endoplasmic reticulum calcium channel. (**C**) Fe homeostasis, intracellular transporters, and possible α-syn interactions. Fe enters via transferrin/transferrin receptor or via DMT1 and can leave via ferroportin. Endocytosed ferritransferrin releases Fe(II) via DMT1. PCBP-1 transports Fe to be stored in ferritin. Fe accumulation may lead to interactions, such as with α-syn or MT. Fe release from ferritin and/or transferrin may also be inhibited by α-syn. PCBP-1/2 induction, such as by Meis1, may combat Fe accumulation. DMT1, divalent metal-ion transporter 1; PCBP1, poly (rC)-binding protein 1. Dashed arrows indicate tentative pathways.

Labile Cu within certain fluids or regions has a propensity to cause protein aggregation and lead further toward a disease state. In the brain, ATP7A/B in the trans-Golgi membrane receive Cu from the Cu chaperone, antioxidant 1 copper chaperone (Atox1), from where it may enter secretory vesicles. Cu can induce the aggregation of α-syn via high-affinity N-terminal and low-affinity C-terminal sites [23]. As the N-terminus of α-syn spans the vesicular membrane (Figure 1), this may provide a mechanism for exposure of the high-affinity Cu-binding site to the intralumenal Cu pool. It is Cu in the mislocated, or loosely bound form, which potentially causes most harm in PD as it is able to bind to α-syn and induce protein aggregation [24]. Extracellularly, Cu is present mainly as Cu(II) but enters cells via the CTR1 transporter as Cu(I), so must be reduced before uptake. It is likely to be present as Cu(I) intracellularly except when bound in stable coordination environments, such as in cuproproteins, where the Cu(II) oxidation state may be stabilized. Cycling between Cu(I) and Cu(II), such as under conditions of oxidative stress, may further contribute to the formation of reactive oxygen species (ROS) (Figure 2). Cu is buffered intracellularly by copper-binding proteins, such as metallothionein (MT) [9]. Indeed, the binding affinity of Cu(I) to MT is estimated to be approximately 12 orders of magnitude greater than that of α-syn [23,25,26]. Although binding of Cu(I) to MT is essentially irreversible, an apparent Kd of ~10^−19^ M has recently be determined [26], compared to estimates for α-syn: Cu(I) of ~10^−6^ M, Cu(II) of 10^−9^ M for Cu(II) in site I and 10^−8^ M for site II [23]. Binding of Cu(II) to MT involves reduction to Cu(I) with concomitant oxidation of cysteine residues to form cysteine disulfides. This means that any Cu entering the cytosol not directed to specific partner proteins is likely to be bound by MT in preference to α-syn. Recent studies have shown that the Cu-dependent superoxide dismutase, SOD1, may be under-metallated in PD [27]. Cu transfer to SOD1 occurs via the specific Cu transporter, Cu chaperone for superoxide dismutase (CCS), rather than via Atox1. Indeed, a mutation in CCS that disturbs SOD1 metallation has been linked to familial forms of amyotrophic lateral sclerosis (ALS) [28], which is characterized by similar SOD1 aggregate pathology as reported recently in PD [29,30]. Although the different pathway taken by Cu entering the vesicular system via Atox1 compared to CCS-mediated loading of Cu into SOD1 may account for potential differences in the interactions of Cu with α-syn and SOD1 in PD, it remains unclear how MT could potentially intercept Cu as there is evidence that Cu is transferred directly from CTR1 to membrane-associated CCS (or likely also Atox1) [31]. MT likely only binds Cu when Cu is outside these pathways or when Cu enters the cell other than via CTR1. Both MT and α-syn are also secreted by brain cells by mechanisms that remain unclear. Therefore, a similar competition for Cu between MT and α-syn will also exist in the extracellular space, although the scope for Cu(I)/Cu(II) cycling will be greater and the concentrations of extracellular MTs are not known. Furthermore, extracellular MT can be taken up via the Lrp1/2 transmembrane transporter [32] and extracellular α-syn has been found to interact with a variety of cell surface receptors that can mediate endocytosis [7].

## 3. Induction of Metallothionein as an Anti-Copper Therapeutic

MTs act to regulate levels of metals in the body by efficient binding and sequestration. MTs function to augment the homeostatic mechanisms of Cu, Zn, and possibly Fe and could also function as ROS absorbers in the CNS [33]. Evidence is accumulating to support the capacity of MTs for neuroprotection against proteinopathy-driven neurodegenerative conditions, such as PD and Alzheimer’s disease. Some studies indicate that MT-3, the brain-specific isoform with higher Cu(I) affinity than MT1/2, is decreased in disease [34,35,36,37,38]. Recently, studies have shown that MT induction by the glucocorticoid analogue, dexamethasone, could block Cu-dependent α-syn aggregation [39]. Figure 2A summarizes the regulation of intracellular Cu, interactions between Cu, α-syn, and MT and the induction of MT by dexamethasone. Although MT binds Cu avidly, increased MT expression may also exert a neuroprotective action by efficiently scavenging intracellular ROS generated by Cu(I)/Cu(II) redox cycling. MT levels also respond to the concentration of intracellular metal ions via the metal-dependent transcription factor-1 (MTF-1) [40]. In the brain, MT-1/2 is strongly expressed by astrocytes and has been shown to be elevated in astrocytes in post-mortem tissue of PD cases [41]. While protoplasmic astrocytes in PD do not show a typical reactive phenotype, they do display abnormal α-syn accumulation [42]. Astrocytic expression of α-syn is associated with the induction of neurotoxic microglia, which leads to neurodegeneration [43]. Therefore, elevating the neuroprotective and anti-oxidant potential of astrocytes via MT induction may hold therapeutic value. MT secretion may also enable suppression of extracellular ROS production due to raised Cu levels in the extracellular space. Although chronic administration of dexamethasone or other glucocorticoid analogues may not be appropriate therapeutically, other MT inducers with less well-defined targets on the MT promoter, such as the food additive, β-thujaplicin, and the cGMP-specific phosphodiesterase type 5 inhibitor, sildenafil, have been described [44,45]. One limitation of this approach may be the possible side effects of MT induction in unintended cell types. Ideally, it would be desirable to design cell-type specific targeting of a potentially therapeutic MT inducer for PD treatment.

Increased brain MT expression is also observed in the atypical Parkinson’s disease, multiple system atrophy, and is associated with pathological α-syn aggregates. The heightened MT levels, notably MT-3, seen here were theorized to be a result of protective upregulation in the brain [46]. Indeed, MT-3 appears to have particular neuroprotective capacity against Cu-bound amyloid [47]. Overall, it is clear Cu dysregulation, which is observed in PD, may result in labile Cu in several fluids and cellular compartments. This may in turn lead to accelerated α-syn aggregation and contribute to PD disease progression. Cu in the extracellular fluid, being present as Cu(II), may be of particular importance due to the production of ROS and through interaction with extracellular α-syn (Figure 2A), although extracellular α-syn may have greater relevance in atypical PD variants, such as multiple system atrophy and dementia with Lewy bodies [48]. It has also been suggested that the apparent under-metallation of SOD1 observed in PD may be due to an under-supply of Cu and that this may be overcome by Cu supplementation therapy [27]. Much as targeting of potentially therapeutic MT inducers to specific cell types may be required to avoid unwanted side effects, it may also be advantageous to induce MT biosynthesis in tandem with Cu supplementation to block the damaging effects of excess Cu supplied via non-cell-type-specific drugs such as CuATSM.

## 4. Ca Dysregulation in Parkinson’s Disease

Initial studies by Yamada et al. (1990) provided the first indication of a role for Ca in PD pathogenesis, finding that dopaminergic neurons of the Sn that express detectable levels of the vitamin D-dependent calcium buffering protein, Calbindin-D28k (CB), were preferentially spared in PD patients compared to control cases. These data suggested that an important factor in the pathogenesis of α-synucleinopathies could be the low Ca(II) buffering capacity of vulnerable neurons [49]. There is no evidence that resting intracellular Ca levels increase with age. However, in aged neurons, the return time to resting levels is believed to be significantly increased after a stimulus [50]. Initiating cell death via the intrinsic pathway, α-syn oligomers have been shown to induce mitochondrial depolarization, inhibit mitochondrial complex I, and activate Ca signaling [51]. Oligomeric α-syn can also mediate the influx of Ca(II) [52]. Furthermore, the Isradipine Ca-channel blocker found to be neuroprotective in PD models that is now in phase III PD clinical trials (STEADY-PD) abrogated Ca-dependent mitochondrial oxidative stress [53]. Oligomeric forms of α-syn were found to bind and activate the endoplasmic reticulum calcium pump (SERCA) in vitro leading to Ca dysregulation [54]. Altered ER Ca control has also been reported in LRRK2^G2019S^ PD iPSC-derived neurons [55]. Moreover, α-syn can interact with Ca(II) influx to mediate increased oxidative stress [56,57] and raised intracellular free Ca(II) and oxidative stress combined synergistically to promote the intracellular aggregation of α-syn [58]. Indeed, incubation of α-syn with Ca in the presence of hydrogen peroxide induced the formation of intramolecular dityrosine cross-links [59] and led to the formation of stable tetramers that surface assemble as annular oligomers [58]. Furthermore, Ca binding to α-syn was found to stimulate membrane binding and induce vesicle clustering with Ca binding to α-syn with Kd = 21 µM [60]. Indeed, recent pathological studies showed that, in the atypical Parkinsonian syndrome, dementia with Lewy bodies, α-syn aggregates were almost completely absent from CB-positive neurons in multiple brain regions. In the same study, analysis of a mouse model of PD that employed unilateral lesion with the mitochondrial complex I inhibitor, rotenone, demonstrated similar exclusion of α-syn inclusion bodies from CB-expressing neurons in the lesioned hemisphere [61,62]. Recently, Mosharov and co-workers found that the increased 1-methyl-4-phenyl-1,2,3,6-tetrahydropyridine (MPTP) sensitivity of Sn neurons was related to α-syn-dependent Ca uptake [63]. The mechanism of rotenone-mediated α-syn aggregation has also recently been linked to its ability to mediate raised intracellular Ca, whereby the calcium/GSK3beta signaling pathway was implicated in rotenone-induced α-syn aggregation and intracellular Ca chelation was shown to ameliorate rotenone-induced impairments of autophagy [64]. More recently, Ca was shown to increase the lipid binding of α-syn to isolated synaptic vesicles via its N terminus and C terminus. Indeed, Ca mediates the localization of α-syn at the pre-synaptic terminal, and an imbalance in Ca can cause α-syn aggregation ex vivo and in vitro [65]. 

## 5. Calbindin D-28k Induction as A Neuroprotective Strategy in Parkinson’s Disease

Ca homeostasis is key to numerous important pathways that maintain healthy cellular function and is closely regulated by endoplasmic reticulum and mitochondrial stores and via several specific proteins that modulate Ca buffering in neurons, including CB, calretinin, and parvalbumin [65]. Work by German and co-workers on idiopathic PD and on MPTP monkey and mouse models illustrated the vital importance of Ca buffering proteins, showing that, in CB-negative regions, neurons were lost preferentially compared to neurons that were CB-positive, which were relatively spared [66]. Reduced levels of CB mRNA in the dentate gyrus were found in the post-mortem brain tissues from patients with dementia with Lewy bodies [67]. Furthermore, relative sparing of Sn dopamine neurons containing CB was observed in PD patients [49]. Indeed, Yuan and co-workers found that mice overexpressing CB were characterized by reduced neuronal loss in an oxidative stress PD mouse model [64]. Recently, it was shown that treatment with the vitamin D analogue, calcipotriol, resulted in a dose-dependent increase in CB expression in SH-SY5Y neuroblastoma cells. Furthermore, calcipotriol suppressed cytoplasmic α-syn aggregate formation promoted by raised intracellular Ca(II) by a CB-dependent mechanism [68]. This indicates that, by promoting the expression of CB at the transcriptional level, calcipotriol was able to target raised neuronal intracellular free Ca(II) and inhibit α-syn aggregation. With a dissociation constant for Ca(II) ions of 393 nM [69], CB binds calcium almost two orders of magnitude more avidly than α-syn. The Ca(II) concentration in resting neurons is ~100 nM, so Kd values of both CB and α-syn are well above the homeostatic level, with both proteins exhibiting properties of calcium sensors, becoming occupied only during short-lived neuronal calcium transients [65,70]. Furthermore, recent studies have shown that virus-vector-mediated neuronal targeting of CB expression could ameliorate neurodegeneration in an MPTP mouse model of PD [71]. Figure 2B summarizes the regulation of intracellular Ca and the interaction with α-syn, highlighting the potentially therapeutic induction of CB transcription.

## 6. Fe Accumulation in Parkinson’s Disease

Fe is important for oxygen transport by red blood cells, as a cofactor for enzymes, such as aconitase and catalase, and in electron transport proteins. Sporadic and familial PD is associated with Fe dyshomeostasis in the Sn. Fe levels within the midbrain that are abnormally elevated compared to normal ageing is a pathological feature of PD, and it has been suggested that neurodegenerative processes observed in PD may be initiated by aberrant reactions between dopamine metabolites and redox-active Fe(II)/Fe(III) as a result of defective regulatory and/or anti-oxidant pathways [72]. Neuronal Fe may overwhelm the mechanisms for Fe storage, thereby releasing it to the cytoplasm, where it could interact with dopamine oxidation products not contained within secretory vesicles to form toxic metabolites [73]. Overexpression of α-syn promotes neuronal Fe accumulation, while Fe can also promote aggregation of this protein in vitro and post-translational modifications of α-syn have also been found to regulate Fe transport [74]. Indeed, the membrane-bound tetrameric form of α-syn has been found to show ferrireductase activity that was reduced in PD brain tissue [75]. Both neurons and microglia are known to accumulate Fe, and the severity of PD symptoms correlates with the extent of Fe accumulation [76,77]. Recent studies have indicated that microglial Fe accumulation may promote a senescence-associated secretory phenotype capable of inducing neuronal α-syn aggregation [78]. Ceruloplasmin (Cp) is a multicopper containing ferroxidase involved in multiple physiological pathways including Cu transport, Fe homeostasis, and anti-inflammatory processes [79]. In the brain, Cp is produced by astrocytes and intimately linked to Fe efflux [80,81]. The ferroxidase activity of Cp is dependent upon Cu to catalyze the oxidation of Fe(II) to Fe(III) and subsequent Fe efflux [79]. Additionally, the CSF and Sn of PD patients has decreased ferroxidase activity, which is largely due to Cp, while also containing Cp that has been oxidatively modified [82,83,84]. Therefore, decreased Cu and an increased oxidative environment in the Sn observed in PD could affect the ferroxidase activity of Cp contributing to Fe accumulation. Moreover, increased neuronal Fe may be related to upregulation of the Fe uptake protein, divalent metal transporter (DMT-1), and to downregulation of the Fe export protein, ferroportin. In addition, increased expression of NEDD4 family-interacting protein 1 (Ndfip1), a protein regulating DMT-1, has been reported in the PD, as well as polymorphisms in the transferrin gene [85,86].

The choreographed expression of Fe efflux, uptake and storage proteins that are modulated both transcriptionally and translationally by a set of Fe regulatory proteins maintain cellular Fe homeostasis. Under conditions of Fe overload, ferroportin is upregulated and there is a downregulation of Fe uptake proteins. The principle Fe storage protein, ferritin, maintains homeostatic levels of intracellular Fe by absorbing excess Fe and by mobilizing stored Fe as needed. Nuclear Receptor Coactivator 4 (NCOA4) mediates translocation of ferritin to the autophagophore, which subsequently fuses with the lysosome, wherein the lysosomal cargo, including ferritin, is degraded by lysosomal hydrolases and the liberated Fe moves to the cytoplasm [87]. As the activity of various rab effector proteins, such as Rab1a, are inhibited by α-syn oligomers, thereby resulting in reduced functioning of the lysosome-autophagy system, there will also be an abrogation of ferritin turnover. Indeed, recent studies have linked Fe accumulation to a novel type of cell death pathway distinct from pathways such as apoptosis, named ferroptosis [72], underlining the importance of Fe dysregulation in neurodegeneration.

The amount of Fe to be stored determines the expression of ferritin via the regulation of ferritin biosynthesis. This requires the coordinated action of iron responsive elements (IREs) and IRE-binding proteins (IRPs) that modulate translational efficiency. Indeed, the α-syn gene has been found to contain an IRE [88]. Ferritin is a large, multisubunit protein that is able to accommodate up to 4000–5000 Fe atoms within its core. Cellular Fe uptake results in the initial binding of Fe atoms to the cores of ferritin molecules that are not yet fully saturated with Fe. In concert with this filling up of existing ferritin stores, IRPs are dissociated from ferritin mRNA and that in turn results in increased translation and synthesis of new ferritin molecules. As Fenton chemistry involving the redox cycling of free Fe(II)/Fe(III) ions would otherwise promote the production of reactive oxygen species (ROS), the sequestration of Fe by ferritin also effectively detoxifies the metal, suppressing cell damage. It was shown recently both in the outer retina and using retinal-pigment-epithelial (RPE) cells in culture that α-syn inhibits the lysosome–mediated degradation of ferritin (ferritinophagy), resulting in the intracellular build-up of ferritin and consequently of Fe. Rab1a over-expression was able to restore ferritinophagy, indicating that α-syn blockade of lysosomal function by interfering with the translocation of lysosomal hydrolases via the endomembrane system could cause Fe accumulation as a consequence of reduced ferritinophagy [89].

## 7. Could Inducing Endogenous Iron Chelators Be A Therapeutic Option?

As ferritin expression is regulated in response to Fe load, chemicals that target the ferritin gene promoter could have the potential to chelate excess Fe in PD. However, ferritin is upregulated in activated microglia, and both activated microglia and increased ferritin expression in microglial cells has been observed in PD [76,90,91]. Moreover, as the release of Fe from ferritin stores occurs through the autophagic degradation of ferritin protein, agents that boost biosynthesis of ferritinophagy components, such as Rab1a, could lead to reduced intracellular Fe deposition. In addition to the role of ferritin in intracellular Fe storage, Fe uptake can be mediated by the extracellular Fe shuttle, transferrin, and by DMT-1 [92]. Transferrin containing Fe(III) interacts with the cell-surface transferrin receptor and is endocytosed. Once inside the cell, passage of ferritransferrin through endosome compartments results in reduction to Fe(II) by STEAP3 and the acidification of the late endosome causes release of Fe(II), which is exported to the cytoplasm via DMT1, whereupon apo-transferrin is returned to the cell surface and released to the extracellular space. Extracellularly, Fe(II)/Fe(III) redox cycling can efficiently generate ROS by the classical Fenton chemistry.

Interaction between α-syn and components of the endomembrane system could inhibit transferrin transit, thereby resulting in transferrin accumulation and further increasing the accumulation of intracellular Fe. Fe(II) can bind to both MT and α-syn in vitro, although with low affinity, and the redox chemistry of Fe may also contribute to α-syn aggregation via ROS production. Indeed, recent studies have indicated that Fe can promote α-syn aggregation and transmission by inhibiting autophagosome-lysosome fusion mediated by transcription factor EB [93]. The passage of Fe to ferritin after the release by transferrin involves the Fe binding proteins, poly(C)-binding protein 1/2 (PCBP-1/2) [92,94]. PCBP-2 also promotes Fe efflux via the sole iron export transporter, ferroportin [92]. Meis1, a member of the TALE (three amino acid loop extension) family of homeodomain transcription factors, was found recently to induce transcription of PCBP-2 [95] and Meis1 overexpression inhibited angiotensin II activation of the Akt-mTOR pathway. Figure 2C outlines the interactions of Fe(II)/Fe(III) that may be relevant to PD. It is tempting to speculate that synthetic angiotensin II receptor effectors or modulators of Meis1 may be able to upregulate the expression of PCBP1/2 and influence the intracellular Fe equilibrium.

## 8. Potential for Novel Approaches to Metal Dysregulation in Parkinson’s Disease

The role of metal ions in the pathogenesis of PD, in particular those of Cu, Ca, and Fe, has attracted considerable recent attention. Indeed, Dare et al. (2017) have suggested that excessive juvenile exposure to dietary Fe could be a major contributor to elevated brain Fe and represent a significant risk factor for PD [96]. There are currently no mechanism-based neuroprotective drugs to treat PD. We have highlighted recent studies that suggest that transcriptional inducers, such as vitamin D analogues and synthetic glucocorticoids, may be novel mechanism-based drugs against metal dysregulation in PD. Further research is needed to determine suitable candidates where metal chelation therapy for Cu, Ca, or Fe may be useful. The application of the induction of endogenous metal regulatory proteins, rather than chemical metal chelators such as deferiprone, may offer a potentially less toxic approach to treating PD and related diseases.

## Abbreviations

α-syn	α-synuclein
CNS	central nervous system
CSF	cerebrospinal fluid
LB	Lewy body
PD	Parkinson’s disease
DMT1	divalent metal ion transporter 1
MT	metallothionein
CB	calbindin-D28k
PCBP-1	poly(C)-binding protein 1
CTR1	Cu transporter 1
Sn	substantia nigra
